# UPLC-ESI-MS/MS and HPTLC Method for Quantitative Estimation of Cytotoxic Glycosides and Aglycone in Bioactivity Guided Fractions of *Solanum nigrum* L.

**DOI:** 10.3389/fphar.2017.00434

**Published:** 2017-07-06

**Authors:** Karishma Chester, Sarvesh Paliwal, Washim Khan, Sayeed Ahmad

**Affiliations:** ^1^Department of Pharmacy, Banasthali UniversityTonk, India; ^2^Bioactive Natural Product Laboratory, Department of Pharmacognosy and Phytochemistry, Faculty of Pharmacy, Jamia Hamdard UniversityNew Delhi, India

**Keywords:** *Solanum*, enriched fraction, steroidal glycosides, quantification, hepatoprotection

## Abstract

*Solanum nigrum* L., is traditionally used for the management of the various liver disorders. Investigating the effect of polarity based fractionation of *S. nigrum* for its hepatoprotective effect on Hep G2 cells *in vitro* to provide base of its activity by quantifying in steroidal glycosides responsible for hepatoprotective potential. A new UPLC-ESI-MS/MS method following a high performance thin layer chromatography (HPTLC) has been developed and validated for quantification of steroidal glycosides and aglycone (solasonine, solamargine, and solasodine, respectively). The *in vitro* antioxidant potential, total phenolics, and flavonoid content were also determined in different fractions. The newly developed UPLC-ESI-MS/MS and HPTLC methods were linear (*r*^2^ ≥ 0.99), precise, accurate, and showing recovery more than 97%. The n-butanol enriched fraction of *S. nigrum* berries was found to be the most potent hepatoprotective fraction against all other fractions as it showed significantly (*p* < 0.01) better *in vitro* anti-oxidant potential than other fractions. Quantification by both methods revealed that, content of steroidal glycosides and aglycones are more than 20% in n-butanol fraction as compared to other fractions. The screened steroidal glycoside n-butanol enriched fraction underwent bioefficacy studies against D-galactosamine and H_2_O_2_ induced toxicity in HepG2 cell line showing significant (*p* < 0.05) liver protection. However, developed method can be used for the quality control analysis with respect to targeted metabolites and it can be explored for the pharmacokinetic and pharmacodynamic analysis in future.

## Introduction

In western and developing countries, liver disorders including liver cirrhosis and drug induced liver injury are the leading cause of death. Currently, available treatment in modern medicine for hepatic disease are often carry the risk of adverse effects, and are often too costly (Jain S. K. et al., 2013). Plants are well-known traditional medicines which are being used since decades for the management of liver disorders. Thus, treatment of liver diseases with plant-derived compounds seems to be highly attractive because of their accessibility and easy to isolate (Sagar et al., 2014). Herbal medicines have been used to treat liver disorders have now become a promising therapy hence it plays a key role, where 65% of patients in US and Europe depend on herbal preparations for the treatment of liver diseases (Zhang et al., 2013).

*Solanum nigrum* L. a traditional Ayurveda plant, used for centuries having wide spectrum of pharmacological properties such as antioxidant, anticancer, hepatoprotective (Abd Elkawy et al., 2013; Abdel-Rahim et al., 2014), neuroprotective and antiulcerogenic (Arulmozhi et al., 2012). The plant has been extensively used in several Ayurveda and herbal formulations recommended for hepatoprotection such as Evecare®, Lifur®, liv 52®, Herbolax®, and Jaunisse® syrup and capsules etc. in India as major component (Sandhir and Gill, 1990). However, the future of the treatment depends on our understanding about the role of chemical constituents of responsible plants (Rajaratnam et al., 2014).

The pharmacological actions of solamargine and solasonine are to treat various types of cancer such as glioblastoma multiform, liver, colon, bladder, rectal, basal cell and squamous cancers, lung cancer, and other respiratory cancers (WEB_3, 2006). Furthermore, solamargine and solasodine reveal cytotoxicity to human liver cancer cells through apoptosis (Cheng et al., 1998). However, solasodine, solamargine, and solasonine from *S. incanum L*. showed hepatprotective activity against ethanol and CCl_4_-induced liver damage (Lin et al., 1990; Liu et al., 2016). All the above mentioned biological activities focused on the steroidal glycosides as major constituent of the Solanaceae family, suggesting that the above constituents are the principle bioactive compounds existing in *S. nigrum*. The desire to maximize the potentiality of phytochemicals has led to the development of bioactive enriched fractions containing higher amount of metabolite of interest. Although, enriched bioactive fraction effectively increases the bioactivity of the extract but its standardization and quality control process can be challenging (Imam et al., 2016). In traditional system of medicine, *S. nigrum* has been used for various diseases especially in liver disorders. In support of its traditional claim, researchers have investigated the effect of its various extract against liver disorders and steroidal glycosides are found as responsible bioactive molecules. In our present study, we developed a method for isolation of steroidal glycosides enriched fraction from *S. nigrum* and which can be used as therapeutic medicine as parallel to modern system of medicine. Recently, glycoalkaloids from *S. nigrum* drawn a keen interest and possess various therapeutic potential (Abbas et al., 1998). The major steroidal alkaloids are solanine, solasonine, and solamargine are the few major steroidal alkaloidal glycosides (SGAs) found in egg plants (*S. melongena*) and in at least 100 other Solanum species (Blankemeyer et al., 1998). Structurally, these three glycoalkaloids have the same steroidal part of the molecule (aglycone), solasodine, but differ in the nature of the carbohydrate side chain.

As far analysis concern, very few methods has been developed for the quantification of steroidal glycosides which includes estimation of solamargine and solasonine by HPLC and HPTLC (Tiossi et al., 2012; Sinani et al., 2015), solasodine by LC-MS and HPLC (Thongchai et al., 2011; Ma et al., 2014). Till present, to our best knowledge, there are no such simultaneous method for quantitative determination of solamargine, solasonine, and solasodine as major bioactive constituents of our interest. Because of diversity of type of chemistry among these three metabolites (solamargine, solasonine, and solasodine), it is very difficult to quantify these metabolites simultaneously. Herein we report the methods for simultaneous estimation of solamargine, solasonine, and solasodine through HPTLC and by UPLC-ESI-MS/MS. HPTLC method can be used for quality control analysis and quantitative evaluation also. Whereas, UPLC-ESI-MS/MS method can be used to quantify these metabolites in plasma to characterize its pharmacokinetic property and to understand its pharmacological features. These two analytical methods are simple, accurate, sensitive, and reliable and could be used to analyze varied amounts of solasmargine, solasonine, and solasodine in plant extract and in rat plasma.

## Materials and methods

### Procurement of plants and chemicals

Plant material collected from local fields of Aligarh (India) and was authenticated in Bioactive Natural Product Laboratory, Jamia Hamdard as per protocol described in Ayurveda pharmacopeia (Anonymous, 2014). Further, voucher specimen of same was deposited in Herbarium of BNPL (Specimen No.: JH/FP/BNPL/2014/Karishma/S1) for future reference. Analytical markers, solasodine, solamargine, and solasonine were procured from Glycomix (Whiteknights Road, UK) and were used as reference. Roswell Park Memorial Institute medium (RPMI-1620) (Gibco, USA), Fetal bovine serum (FBS) (Gibco, USA), Trypsin-EDTA (Sigma-Aldrich, USA), trypan blue (Sigma-Aldrich, USA), Phosphate buffer saline (PBS) (Gibco, USA), penicillin-streptomycin solution (Sigma-Aldrich, USA), DMSO (Dimethylsulfoxide) (Sigma–Aldrich, USA), 2,2-diphenyl-1-picrylhydrazyl (DPPH, 0.002%, w/v). Other chemicals and reagents used were of analytical grade (AR) and procured from Merck Ltd., India.

### Preparation of hydro alcoholic extract (mother extract) and its fraction

The 500 g of powdered *S. nigrum* ariel parts (leaves and berries, separately) was shade dried and extracted with 70% ethanol (v/v) in reflux extractor for 5 h. The extract was filtered and filtrate was evaporated to dryness under reduced pressure. The hydroalcoholic (mother) extract was suspended in double distilled water (1 g/10 mL) and sonicated for 15 min at 45°C. Aqueous suspension was fractionated with equal proportions of hexane, dichloromethane (DCM) and n-butanol. The residue and solvent fractions obtained were evaporated to dryness under reduced pressure. The extractive values and % yields of different fractions were calculated and stored at 4°C for further use.

### HPTLC and UPLC−ESI-MS/MS method of qualitative and quantitative validation for *S. nigrum*

HPTLC analysis of *S. nigrum* extract and its different fractions (hexane, DCM, n-butanol, and water) were carried out for their qualitative and quantitative analysis. Hence, simultaneous analysis of these compounds in the different fractions was carried out using newly developed HPTLC method followed the ICH guidelines (Tambe et al., 2014).

#### HPTLC sample preparation and chromatographic conditions

The dried mother extract and fractions (100 mg each) of *S. nigrum* were reconstituted using methanol (HPLC grade) and prepared 10 mg/mL concentration of the extract and fraction. The samples were applied on pre-coated HPTLC silica gel aluminum plates (60F_254_; 20 × 10 cm, Merck KGaA, Germany) in 4.0 mm width band length with a CAMAG microlitre syringe using a CAMAG Linomat V (Muttenz, Switzerland) and were controlled by WinCATS software (CAMAG). A constant application flow rate of 100 nL/s was directed and the space between two bands was 7.2 mm. The slit dimension was maintained at 5.0 × 0.3 mm, and 20 mm/s scanning speed was employed. The solvent system of the SGAs consisted of n-butanol: ethyl acetate: acetic acid 10%, (5:3.5:1.5, v/v/v). Linear ascending development was carried out in a 20 × 10 cm twin trough glass chamber, saturated with the solvent system. The optimized chamber saturation time for the solvent system was 15 min at room temperature. The length of chromatogram run was 80 mm. Subsequent to development, HPTLC plates were dried in an oven at 60°C for 5 min. Densitometric scanning was performed on a CAMAG TLC scanner IV (absorbance mode 530 nm) with WinCATS software after spraying the developed plate with anisaldehyde-sulphuric acid reagent and heating it on a hot air oven at 110°C for 5 min.

#### UPLC-ESI-MS/MS sample preparation and chromatographic conditions

Quantitative analysis was performed on a Waters Acquity UPLC H-Class-Xevo TQD system (MA, USA) equipped with electrospray ionization operated in the positive ionization mode. Chromatographic separation of analytes was carried out on Acquity UPLC HSS C18 (50 × 2.1 mm, 1.8 m) column using a gradient mobile phase consisting of 0.1% formic acid (A) and methanol (B) at a flow rate of 0.4 mL/min, under gradient conditions: A:B %v/v (0 min: 95:5; 1.5 min: 10:90; 2 min: 10:90; 3 min: 5−95; 4 min: 10−90; 4.5 min: 95:5; and 5 min: 95:5). The column was maintained at 25°C and the pressure of the system was 6,500 psi. The source dependent parameters maintained for the analytes: cone gas flow: 50 l/h; desolvation gas flow, 800 l/h; capillary voltage, 3.5 kV, source temperature, 120°C; desolvation temperature, 350°C. The optimum values for compound dependent parameters like cone voltage and collision energy were set at 80–62 kV for solasonine, 66–52 Kv for solamargine, and 74–38 kV for solasodine. Unit mass resolution was employed and the dwell time was set at 0.75 ms. Detection of the ions was performed in the multiple-reaction monitoring (MRM) mode, by monitoring the transition pairs (precursor to product ion). Mass Lynx software version 4.1 was used to control all parameters of UPLC and ESI-MS/MS. All experiments were performed in triplicate. A stock solution containing identical amounts of compounds was prepared by dissolving the reference compounds in methanol and then diluting it with methanol to appropriate concentrations for achieving calibration curves.

### Validation of HPTLC and UPLC-ESI-MS/MS method

The validation of developed HPTLC method was done as per the ICH guidelines (Tambe et al., 2014) similar to the other chromatographic HPTLC methods reported (Singh et al., 2011; Parveen et al., 2014; Mallick et al., 2015) and confirm the quality control of herbal drugs. However, the developed UPLC-ESI-MS/MS method was validated in order to meet the acceptance criteria of guidance to the industry bio analytical method validation recommended by USFDA and EMEA guidelines (CHMP, 2009).

#### Calibration curve

Different concentration of SGAs mixture (5, 10, 20, 40, 60, 80, and 100 ng/mL) were used for UPLC-ESI-MS/MS while for HPTLC different volumes of SGAs mixture (0.1, 0.2, 0.4, 0.6, 0.8, 1.0, 2.0, 4.0, 6.0, and 8.0 μL/mL) were spotted on the HPTLC plate to obtain the 16.6, 33.2, 66.4, 99.6, 132.8, 166, 332, 664, 996, and 1,328 ng/spot of each SGAs, respectively. The data for the peak area vs. solasonine, solamargine, and solasodine concentrations were treated with the linear-least square regression and the regression equation obtained from the standard curve was used to estimate SGAs in extract.

#### Precision

Inter-day precision analyses were performed via spotting three different concentrations of SGAs stock solution. Inter-analyst precision was carried out by repeating same procedure with a different analyst. Intermediate precisions were determined in terms of %RSD of the area.

#### Accuracy as recovery

The accuracy of the present method was assessed in samples via recovery studies, in which pre-analyzed samples were spiked with additional 50, 100, and 150% of SGAs mixture and analyzed by developed method. The experiments were conducted six times. The average recovered SGAs content was quantified using the regression equation, and the % recovery was calculated accordingly.

#### Robustness

The robustness of developed method was determined at three concentrations in two different ways, i.e., changing the flow rate of mobile phase and changing the percentage of buffer (UPLC-ESI-MS/MS)/ detection wavelength (HPTLC). The %RSD of the peak area was calculated to determine the robustness of the method.

#### Sensitivity

The sensitivity of the method was determined by comparing the limits of detection (LOD) and limit of quantification (LOQ). The concentration of sample giving a signal to noise ratio of three was fixed as LOD, whereas the concentration of sample giving a signal to noise ratio of 10 was fixed as LOQ.

#### Quantification of solasonine, solamargine, and solasodine in different fractions

Both the developed methods were applied to analyse solasonine, solamargine, and solasodine content in different fractions. The SGAs yield was quantified using the regression equation from the calibration curve.

### *In vitro* DPPH radical-scavenging activity and quantitative estimation of total phenolic and flavonoid content

The DPPH radical scavenging activities of *S. nigrum* extract and its fractions were determined according to the previously described method with minor modifications (Wu et al., 2011; Kumar et al., 2014). Briefly, each extract (100–500 g/mL) were mixed with 0.8 mL DPPH solution and incubated in dark room for 1 h. The absorbance was then measured at 517 nm. Ascorbic acid was used as positive control. The DPPH radical scavenging activity was calculated by the following formula:

(1) $$\begin{array}{r} Scavenging\;ability\;\left(\%\right)=\frac{\left(1-A_{sample}\right)}{A_{control}}\times 100. \end{array}$$

Where, *A*_*control*_, Absorbance of control reaction containing methanol instead of sample and *A*_*sample*_, absorbance of test sample.

Hydroalcoholic extract and different fractions were suspended in methanol. In hydroalcoholic extract as well in its fraction the total flavonoid content were determined by using aluminum chloride method (Chang et al., 2002). Whereas, total phenolic content was determined using Folin-Ciocaleteu assay (Esmaeili et al., 2015). Gallic acid (GA) and rutin were used as standards for phenols and flavonoids, respectively and the results were calculated as their equivalents (mg/g) through the calibration curve of gallic acid and rutin, respectively.

### *In vitro* cell culture studies of SGAs

#### Cells and culture conditions

Maintenance of HepG2 cells was done in DMEM medium having 10% FBS and 1x antibiotic-antimycotic solution. Plating of cells was done in 96 well-plates or 60 mm or 100 mm at the density of 1 × 10^4^ cells/mL or 1 × 10^6^ or 3 × 10^6^ plates, respectively for 24 h followed by treatment. Culturing of treated and untreated cells was done in normoxia incubator (Heracell 150i, Thermo Scientific, USA) maintaining 4% CO_2_ at 37°C.

HepG2 cells were maintained in DMEM medium containing FBS (10%) and 1x antibiotic-antimycotic solution. Cells were plated in a density of 1 × 10^4^ cells/mL in 96 well-plates or 1 × 10^6^ cells/mL in 60 mm plates or 3 × 10^6^ cells/mL in 100 mm plates and maintained for 24 h following treatments. Treated and untreated cells were cultured in normoxia incubator (Heracell 150i, Thermo Scientific, USA) maintaining 5% CO_2_ at 37°C in DMEM medium.

#### Treatments

The HepG2 cells was seeded at the density of 1 × 10^4^ cells/well in 96 well-plates and was kept overnight/1 day/ 24 h at room temperature for adherence. For induction of toxicity treatment on cells several concentrations of D-galactosamine was used and considered as toxic control. Further, toxic cells were treated with different concentration of n-butanol fractions of *S. nigrum* berries (0–1,000 μg/ml) and *S. nigrum* leaves (0–1,000 μg/mL) for determination of its protective potential against D-galactosamine induced cytotoxicity. The D-galactosamine was dissolved in ethanol and plant extracts were dissolved in 50–100 μl of DMSO and then in DMEM media. The vehicle controls (ethanol and DMSO) were included in study at appropriate place and did not induce any cytotoxicity or leakage in (alanine aminotransferase) ALAT and (aspartate aminotransferase) ASAT (Reitman and Frankel, 1957). Further, cells were evaluated for bio-efficacy studies for the selected doses from the above mentioned cytotoxicity assays as per previously described protocol (Mallick et al., 2015). Briefly, toxic were plated in a density of 1 × 10^4^ cells/mL in 96 well-plates for 3-(4,5-Dimethylthiazol-2-yl)-2,5-Diphenyltetrazolium Bromide (MTT) and Reactive oxygen species (ROS) assays while cells were grown in 60 mm plates for conducting ALAT and ASAT assays and maintained in incubator for 24 h following treatments.

#### MTT assay

MTT assay was measured by dissolving MTT at concentration of 0.5 mg/mL in culture medium followed by filtering to remove insoluble matter and incubation for 2–4 h following the method of Mosmann (1983) with slight modification. Supernatant was pipette out and extract was be added in a solution of 0.4 N HCl 1: v/v-isopropanol 24 (1:24) v/v. After 25 min of incubation at 30–35°C (room temperature), the absorbance was taken at wavelength of 570 nm on a plate reader (Power Wave XS2, Bio Tek, USA) (Power Wave XS2, BioTek, USA).

#### Assay for intracellular redox state

Measurement of Intracellular redox state was done by using florescent dye, namely 2, 7-dichlorofluorescein di acetate (H2-DCFH-DA). Briefly, washing of cell was firstly done by Hank's Balanced Salt Solution (HBSS) followed by 5–10 μg of DCFH-DA and then incubating for ~30 min at 35–40°C. Reading will be taken at 485 nm and emission at 530 nm (Cary Eclipse, Varian, USA) (Tulsawani et al., 2013).

#### ALAT and ASAT assay

After treatment period, the culture medium was collected and centrifuged to get cell free supernatant for the extracellular ALAT and ASAT estimation. The harvesting of cell was done in sterile phosphate buffered saline (PBS) at pH 7.4 by trypsinization, washing, sonicating for ~10 s. and finally centrifuging at 1,500 g for ~10 min at 4°C. The pellet containing cell debris was discarded and supernatant was used for estimation of intracellular ALAT and ASAT. However, extracellular and intracellular ALAT and ASAT were measured using commercial diagnostic kit purchased from Biodiagnostics Co. (Cairo, Egypt). Leakage of intracellular enzymes was expressed as percent leakage.

### Statistical analysis

All the experiments were performed on three different occasions and data are presented as mean ± SE. The data was analyzed by one-way ANOVA followed by Dunnet's *t*-test for comparing control or hypoxia and the various groups using Graph Pad software. Statistical significance was estimated at the 5% level.

## Results

### Preparation of extracts (leaves and berries) of *S. nigrum*

The plant material was extracted using alcohol: water ratio (70:30, v/v) by reflux extraction after optimization. The extract of the both the sample of the *S. nigrum* were collected separately. Mother extract of berries was further fractionated using hexane, DCM and n-butanol. However, 3.4 g of mother extract (1.0%, w/w) of drug was lost during the processing. Similarly in case of leaves, mother extract was further fractionated using hexane, DCM and n-butanol. However, 5.1 g of mother extract (1.02% w/w) of drug was lost during the processing. The highest extractive value was found in n-butanol's fraction followed by DCM fraction and remaining aqueous fraction. Extractive value in detailed has shown in Table 1.

**Table 1:** Tabular representation of extractive value of mother extract and its fractions of *S. nigrum* (leaves and berries).

**S.no**.	**Fractions**	**Extractive value from mother extract (%w/w)**	
		**Leaves**	**Berries**
1	Hexane fraction	12	8.7
2	DCM fraction	17.7	14.1
3	n-butanol fraction	32	35
4	Remaining fraction	11.4	9.8

### Optimization of the solvent system for HPTLC and various chromatographic condition of UPLC-ESI-MS/MS method

**Figure 1:**
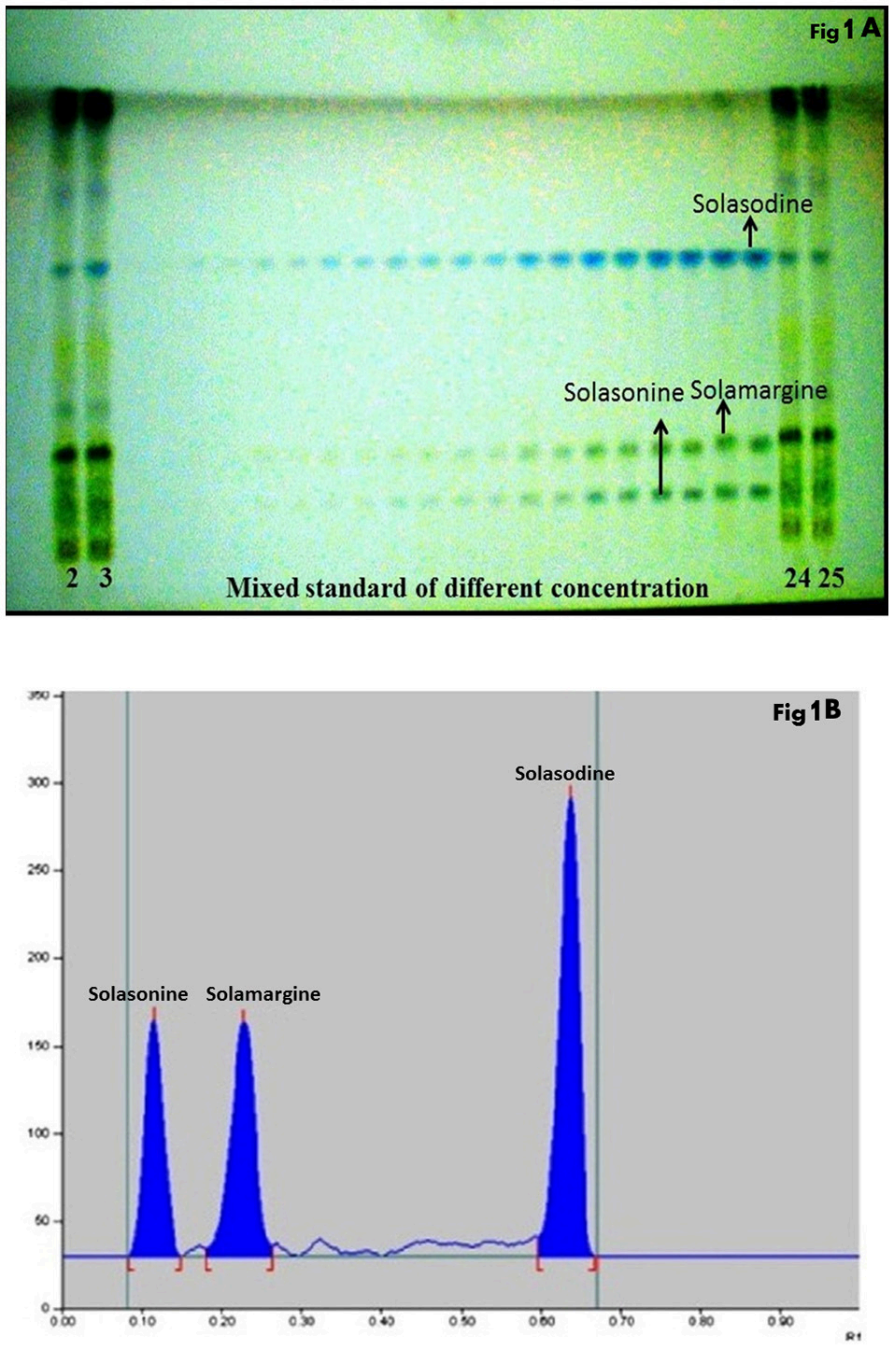
**(A)** Developed HPTLC plate visualized at normal light after derivatisation. **(B)** HPTLC chromatogram of standards solasonine (*R*_*f*_ 0.11 ± 0.02), solamargine (*R*_*f*_ 0.22 ± 0.02) and solasodine (*R*_*f*_ 0.63 ± 0.02) scanned at 530 nm.

**Figure 2:**
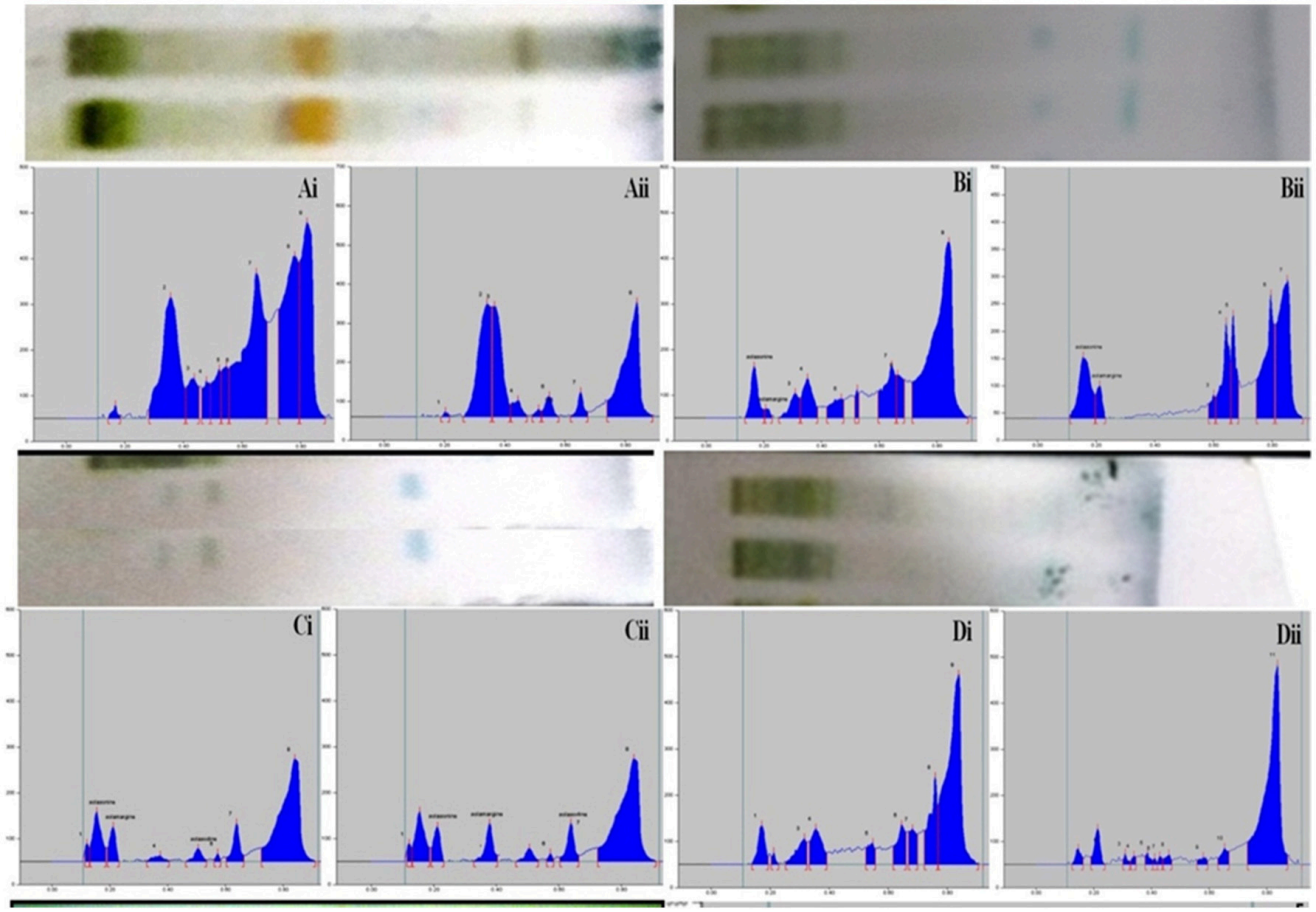
Developed TLC plates and HPTLC chromatograms of different fractions showing peaks of separated markers. Track correspond to **Ai**: Hexane fraction (leaves), **Aii**: Hexane fraction (berries), **Bi**: DCM fraction (leaves), **Bii**: DCM fraction (berries), **Ci**: n-butanol fraction (leaves), **Cii**: n-butanol fraction (berries), **Di**: Final extract (leaves), **Dii**: Final extract (berries).

The composition of the solvent system was optimized by testing different solvent compositions of varying polarities. Different tested compositions of solvent system, the desired resolution of the compounds, together with symmetrical and reproducible peaks, was achieved using the n-butanol: ethyl acetate: acetic acid (5:3.5:1.5, v/v/v) solvent system. Well-separated and compact bands of solasonine, solamargine, and solasodine were visualized at *R*_f_ 0.11 ± 0.02, 0.22 ± 0.02, and 0.63 ± 0.02, respectively using anisaldehyde-sulphuric acid spraying reagent. HPTLC chromatogram of solasodine, soalsonine, and solamargine standard in extract has shown in (Figures 1A,B, 2). Table 2 showing the qualitative analysis of different fractions and identification of solasodine, solamargine, and solasonine.

**Table 2:** HPTLC fingerprint data of mother extract and different fractionation of *S. nigrum* (leaves and berries).

**Extracts/fractions**	**Solvent system**	**Visualization**	**No of major spots and *R*_*f*_ values**	
			**Leaves**	**Berries**
Hydroalcoholic extract of *S. nigrum*	n-butanol: ethyl acetate: acetic acid 10%, (5:3.5:1.5, v/v/v)	Anisaldehyde sulphuric acid at 530 nm	(7): solasonine, 0.17, solamargine, 0.32, 0.40, 0.46, solasodine	(8): solasonine, 0.17, solamargine, 0.23, 0.32, 0.41,0.41, solasodine
Hexane fraction			(3): 0.33, 0.46, 0.50	(4): 0.12, 0.17, 0.32, 0.40
DCM fraction			(7): solamargine, solasonine, 0.36, 0.43, 0.51, 0.55, 0.70	(7): solasonine, solamargine, 0.34, 0.37, 0.44, 0.51, 0.78
n-butanol fraction			(6): solasonine, 0.15, solamargine„ 0.52, solasodine, 0.75	(5): solasonine, 0.15, solamargine„ 0.52, solasodine,
Remaining fraction			(7): 0.16, 0.24, 0.60, 0.64, 0.67, 0.70, 0.75	(5): 0.17, 0.25, 0.33, 0.40, 0.46

**Figure 3:**
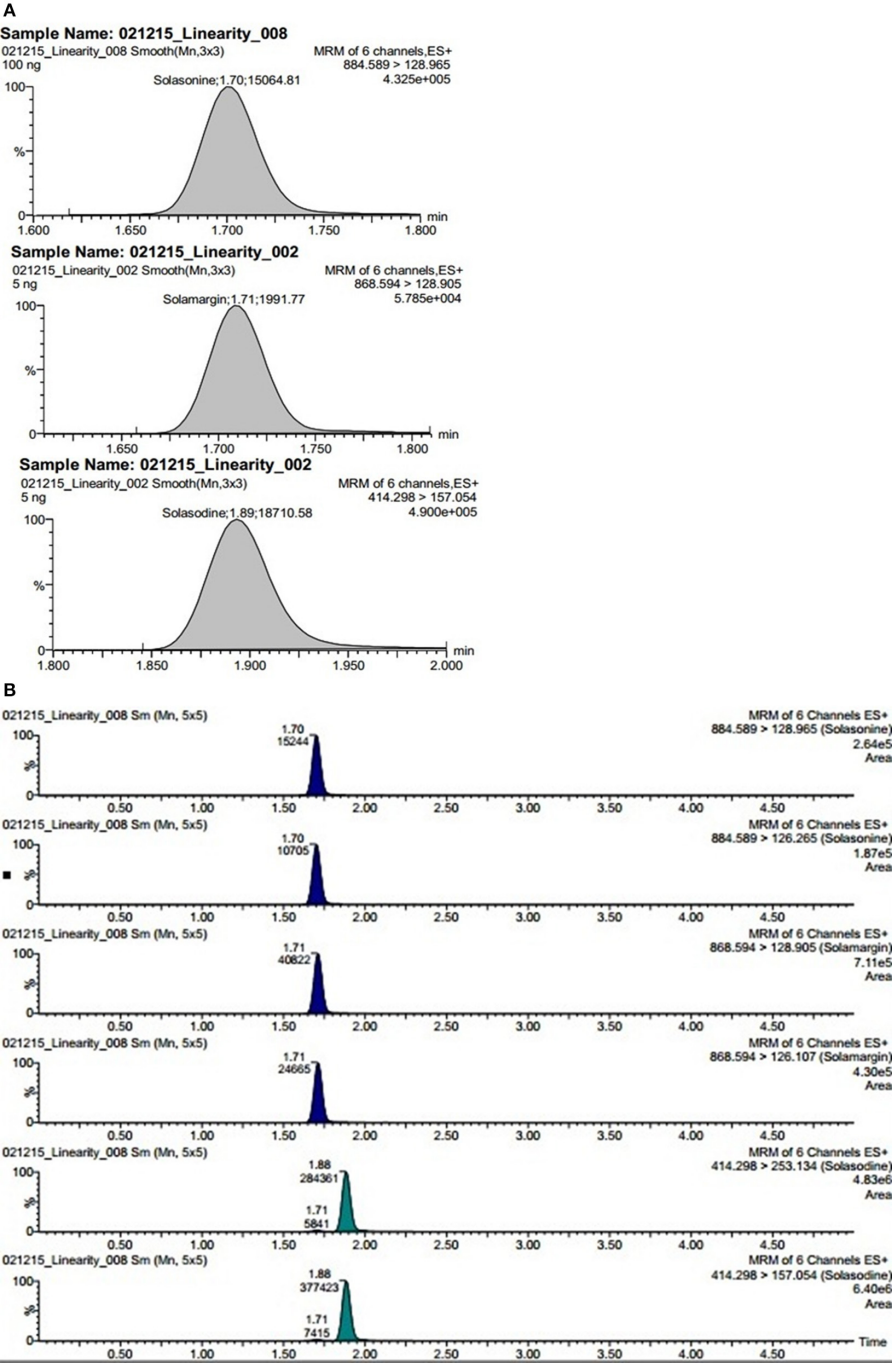
**(A)** Representative chromatogram showing steroidal glycoside and its aglycone namely solasonine, solamargine, and solasodine. **(B)** Extracted ion chromatograms of steroidal glycosides namely solasonine, solamargine, and its aglycone solasodine quantified in plant fraction.

Electrospray ionization in the positive ion mode was used for multiple reactions monitoring (MRM) analysis. The Q1 full-scan mass spectra of solasonine, solamargine and solasodine showed predominant protonated precursor [M + H]+ ions at m/z 884.59 for solasonine, 868.53 for solamargine, 414.30 for solasodine whereas Q2 was set at selective mode at 126.32, 129.02, and 157.05 for solasonine, solamargine, and solasodine, respectively. To optimize the proposed UPLC-MS/MS method, the effects of several chromatographic parameters were investigated. These included the type of organic modifier, pH of aqueous solution, and organic modifier aqueous ratio. These parameters were optimized based on the peak shape, peak intensity/area, and peak resolution and retention time for the analytes (Balcke et al., 2012). Initially, water-methanol was used as an organic modifier along with mobile phase additives like ammonium formate, ammonium acetate, and 0.1% aqueous formic acid. It was observed that the composition and pH of the mobile phase had a significant impact on separation selectivity and sensitivity of the method. The sensitivity was significantly increased with the use of 0.1% aqueous formic acid compared with ammonium acetate or ammonium formate at pH 3.0, using methanol as the organic modifier. Various ratios methanol of 0.1% formic acid were tried to optimize chromatographic separation. Finally, the best chromatographic conditions were achieved using gradient mode of 0.1% formic acid: methanol at a flow rate of 0.4 mL/min (Figures 3A,B).

### Calibration curve for SGAs by UPLC-ESI-MS/MS and HPTLC

The linear regression data for the calibration graph are indicative of a good linear relationship between the peak area and covering a wide range of selected concentrations for both HPTLC and UPLC-MS/MS. The linear regression calibration curves plotted between peak area against the concentration and were linear from 5 to 100 ng/mL for all standard namely solasonine, solamargine, and solasodine with good linear relationships of 0.990, 0.992, and 0.994, respectively, for UPLC-ESI-MS/MS. However, for HPTLC were linear from 166 to 1,328 ng/mL for all standards namely solasonine, solamargine, and solasodine with good linear relationships (*r*^2^ = 0.99) (Table 3).

**Table 3:** Linearity data of chromatographic UPLC-ESI-MS/MS and HPTLC Method for mixed standards (*n* = 3).

**Linearity parameters**	**Solasonine**		**Solamargine**		**Solasodine**	
	**UPLC-ESI-MS/MS (ng/mL)**	**HPTLC (ng/spot)**	**UPLC-ESI-MS/MS (ng/mL)**	**HPTLC (ng/spot)**	**UPLC-ESI-MS/MS (ng/mL)**	**HPTLC (ng/spot)**
Linearity	5–100	16.6–1,328	5–100	16.6–1,328	5–100	16.6–1,328
Equation	Y = 142.53x + 751.37	Y = 179.400 + 1.955x	Y = 348.83x + 4,834.9	Y = 263.823 + 2.080x	Y = 3,477.8x + 32,841	Y = 547.683 + 4.225x
Regression ± *SD*	0.990 ± 0.001	0.997 ± 0.001	0.992 ± 0.0005	0.997 ± 0.0005	0.994 ± 0.0001	0.993 ± 0.0001
Slope ± *SD*	142.53 ± 0.002	179.400 ± 0.002	348.83 ± 0.003	263.823 ± 0.003	3,477.8 ± 0.001	547.683 ± 0.001
Intercept ± *SD*	751.37 ± 0.002	1.955 ± 0.002	4,834.9 ± 0.005	2.080 ± 0.005	32,841 ± 0.001	4.225 ± 0.001
LOD	0.37	2.48	0.33	3.33	0.02	3.89
LOQ	1.15	7.52	1.01	10.09	0.06	11.18

*LOD, limit of detection; LOQ, Limit of quantification; SD, standard deviation; RSD, Relative*.

### Validation of UPLC-ESI-MS/MS and HPTLC method

#### Precision

Intermediate precisions were determined and reported in terms of %RSD. Intermediate precision includes data from inter-day, intra-day, and inter-analyst precision measurements. The satisfactory results (%RSD ≤ 2) of the precision measurements confirm that the method can be used in any laboratory for the estimation of SGAs (Table 4). The acceptance criteria were based on ICH guidelines and accepted practices in industry (Sharma et al., 2017).

**Table 4:** Precision of UPLC-ESI-MS/MS and HPTLC method for estimation of mixed standards (*n* = 3).

**UPLC-ESI-MS/MS**				**HPTLC**			
**Conc (ng/mL)**	**Inter-day precision**	**Intra-day precision**	**Inter-analyst precision**	**Conc (ng/spot)**	**Inter-day precision**	**Intra-day precision**	**Inter-analyst precision**
	**%RSD**	**%RSD**	**%RSD**		**%RSD**	**%RSD**	**%RSD**
**SOLASONINE**							
20	0.46	1.14	0.70	166	1.7	0.79	0.44
40	0.25	7.38	3.53	332	0.11	0.23	0.20
80	0.34	1.7	0.87	664	0.21	0.50	0.52
**SOLAMARGINE**							
20	0.12	3.6	0.37	166	0.16	0.21	0.16
40	0.34	0.07	0.15	332	0.18	0.19	0.10
80	0.45	0.05	0.67	664	0.13	0.17	0.09
**SOLASODINE**							
20	0.02	0.02	0.01	166	0.16	0.14	0.15
40	0.06	0.09	0.04	332	0.10	0.18	0.09
80	0.10	0.07	0.10	664	0.10	0.25	0.08

*Conc. Concentration; RSD, Relative standard deviation*.

#### Accuracy

The method was proved the recovery of the solasonine, solamargine, and solasodine in preanalysed samples were within the range of 97.4–100.1 and 98.9–101.6% for UPLC-ESI-MS/MS and HPTLC method, respectively. 98.27–101.61%, (Table 5). Recovery of metabolites from preanalysed samples were determined by comparing the area of respective peaks of sample with the corresponding peak area of standard solutions.

**Table 5:** Accuracy of UPLC-ESI-MS/MS and HPTLC methods for the estimation of mixed Standards (*n* = 3).

**UPLC-ESI-MS/MS**			**HPTLC**	
**% of standard spiked to the sample**	**% of drug recovered ± *SD***	**%RSD**	**% of drug recovered ± *SD***	**%RSD**
**SOLASONINE**				
0	97.48 ± 0.01	0.03	98.93	0.56
50	100.09 ± 0.04	0.03	98.27	0.16
100	100.16 ± 0.03	0.06	101.61	0.02
150	100.05 ± 0.6	0.02	98.95	0.26
**SOLAMARGINE**				
0	101.11 ± 0.7	0.02	100.13	0.02
50	100.66 ± 0.5	0.1	100.26	0.05
100	100.16 ± 0.4	0.03	100.09	0.02
150	100.15 ± 0.1	0.03	99.92	0.01
**SOLASODINE**				
0	101.76 ± 2.1	0.08	100.39	0.15
50	101.96 ± 1.2	0.04	100.65	0.03
100	101.76 ± 0.7	0.03	100.19	0.02
150	100.84 ± 1.7	0.01	100.24	0.03

#### Robustness

The robustness of the method was assessed by introducing small changes in the composition of % buffer and flow rate (UPLC-ESI-MS/MS), and detection wavelength and solvent system composition (HPTLC), the effect on the desired result was reported as % RSD. The results of robustness measurements were shown in (Table 6).

**Table 6:** Robustness of the UPLC-ESI-MS/MS and HPTLC method for estimation of mixed standards by changing: Buffer % change (*n* = 3), Flow rate (*n* = 3), detection wavelength (*n* = 3), and mobile phase ratio (*n* = 3).

**UPLC-ESI-MS/MS**					**HPTLC**				
**Buffer % change**			**Flow Rate Composition (initial solvent composition A: 0.1% Formic Acid, B: Methanol)**		**Detection wavelength**			**Solvent system composition n-butanol: ethyl acetate: acetic acid 10%, (v/v/v)**	
**Conc (ng/mL)**	**% Change**	**%RSD**	**Ratio change**	**%RSD**	**Conc (ng/spot)**	**Wavelength used (nm)**	**%RSD**	**Ratio change**	**%RSD**
**SOLASONINE**									
20	0.01	0.23	A:B (5:3.5:1.9)	0.11	166	510	0.31	(5:3.5:1)	0.28
	0.05	0.40	A:B (90:0, v/v)	0.16		540	0.35	(5:3.5:1.9)	0.38
40	0.01	0.08	A:B (5:3.5:1.9)	0.14	332	510	0.30	(5:3.5:1)	0.14
	0.05	0.12	A:B (90:0, v/v)	0.07		540	0.23	(5:3.5:1.9)	0.13
80	0.01	0.06	A:B (5:3.5:1.9)	0.03	664	510	0.18	(5:3.5:1)	0.15
	0.05	0.09	A:B (90:0, v/v)	0.08		540	0.08	(5:3.5:1.9)	0.07
**SOLAMARGINE**									
20	0.01	0.09	A:B (5:3.5:1.9)	0.06	166	510	1.50	(5:3.5:1)	0.82
	0.05	0.06	A:B (90:0, v/v)	1.0		540	0.19	(5:3.5:1.9)	0.08
40	0.01	0.02	A:B (5:3.5:1.9)	0.02	332	510	0.13	(5:3.5:1)	0.21
	0.05	0.04	A:B (90:0, v/v)	1.8		540	0.75	(5:3.5:1.9)	0.15
80	0.01	0.02	A:B (5:3.5:1.9)	0.02	664	510	0.10	(5:3.5:1)	0.09
	0.05	0.02	A:B (90:0, v/v)	0.04		540	0.07	(5:3.5:1.9)	0.06
**SOLASODINE**									
20	0.01	0.02	A:B (5:3.5:1.9)	0.02	166	510	0.09	(5:3.5:1)	0.13
	0.05	0.05	A:B (90:0, v/v)	0.02		540	0.3	(5:3.5:1.9)	0.08
40	0.01	0.04	A:B (5:3.5:1.9)	0.02	332	510	0.13	(5:3.5:1)	0.10
	0.05	0.08	A:B (90:0, v/v)	0.03		540	0.13	(5:3.5:1.9)	0.04
80	0.01	0.09	A:B (5:3.5:1.9)	0.04	664	510	0.07	(5:3.5:1)	0.02
	0.05	0.02	A:B (90:0, v/v)	0.06		540	0.14	(5:3.5:1.9)	0.02

#### Sensitivity

Considering the signal intensity at lowest level can be expressed as LOD and LOQ. These parameters represent the concentration of metabolites that would yield signal to noise ratio of 3 and 10 for LOD and LOQ, respectively. For the proposed UPLC-ESI-MS/MS method, LOD and LOQ of solasonine, solamargine, and solasodine were 0.37, 0.33, and 0.02 ng/mL and 1.15, 1.01, and 0.06 ng/mL, respectively. For HPTLC method, LOD and LOQ of solasonine, solamargine, and solasodine were 0.37, 0.33, and 0.02 ng/mL and 1.15, 1.01, and 0.06 ng/mL, respectively.

### Determination of solasonine, solamargine, and solasodine in n-butanol fractions

**Figure 4:**
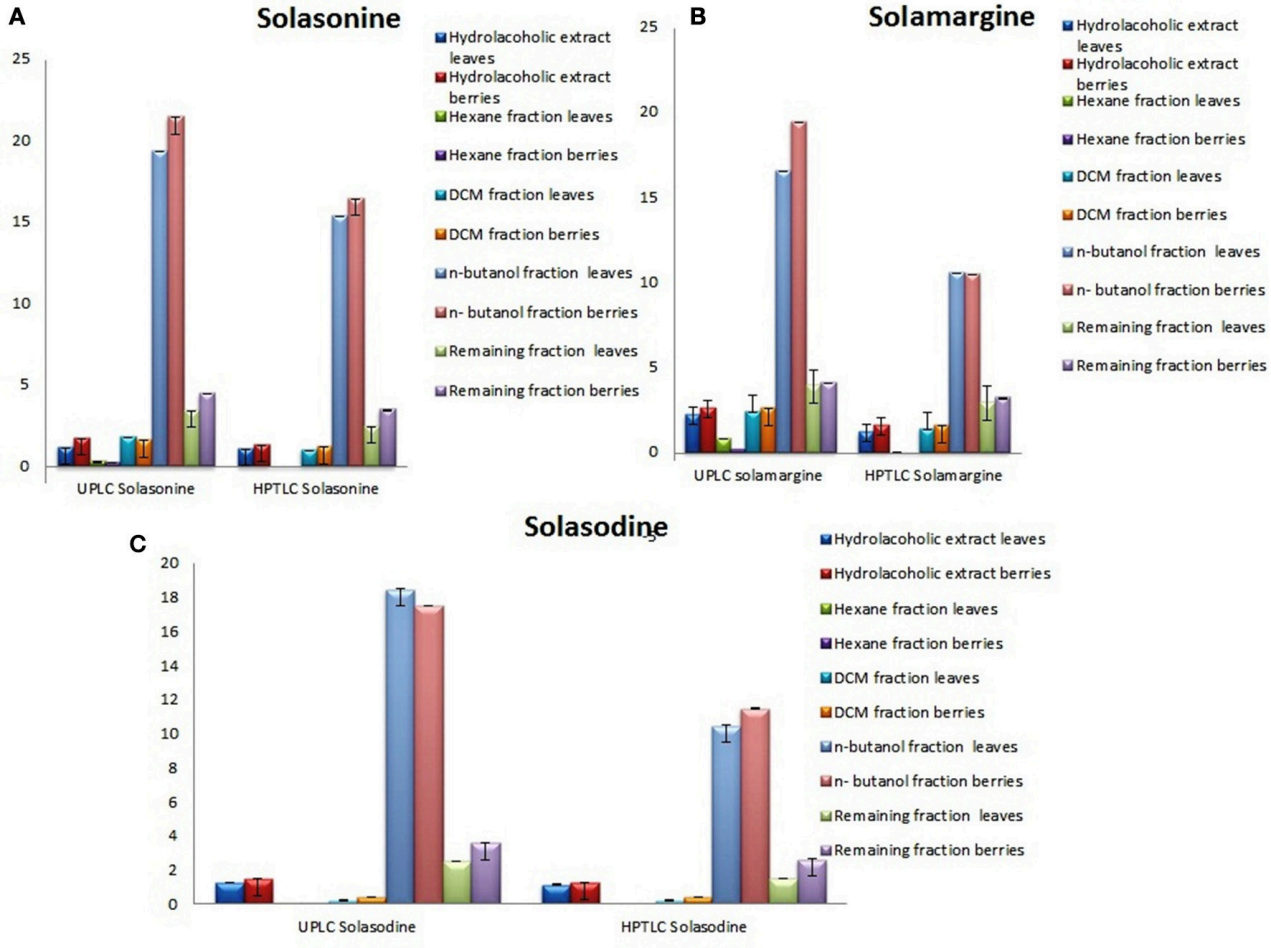
Graphical designing to show the content of **(A)** Solasonine, **(B)** Solamargine, and **(C)** Solasodine present in *S. nigrum* (areil part) mother extract and its fractions by UPLC-ESI-MS/MS and HPTLC analysis.

Showing the glycoalkaloids contents in the aerial part (leaves and berries) of *S. nigrum* fractions are shown in (Figures 4A–C).

### *In vitro* studies of *S. nigrum*

**Figure 5:**
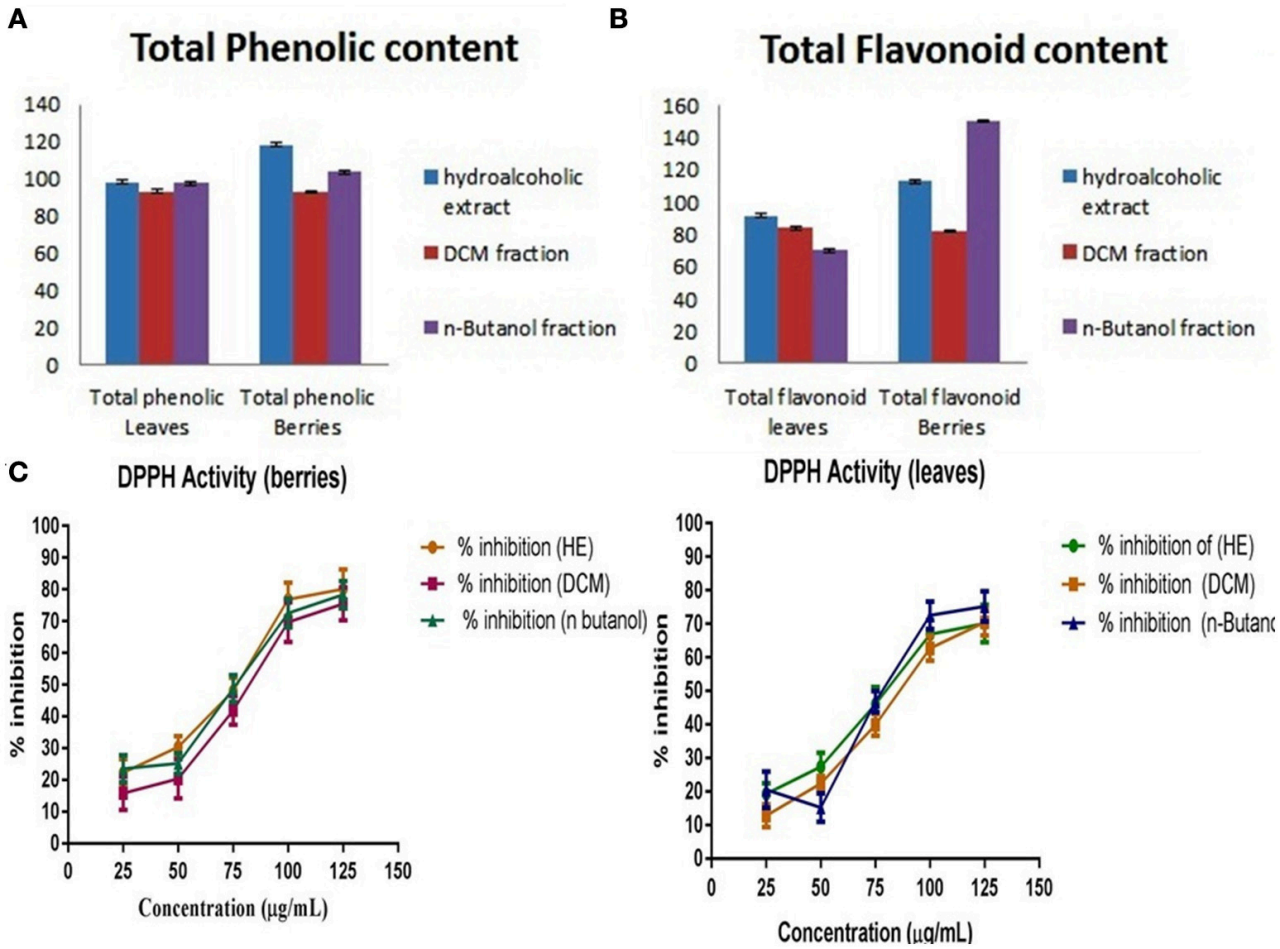
**(A)** Total phenolic content, **(B)** Total flavonoidal contents, and **(C)** Total antioxidant activity of *S. nigrum* (leaves and berries) mother extracts and its fraction.

The quantitative and qualitative estimation of SGAs from *S. nigrum* mother extract and its fraction resulted that hexane and final aqueous fraction were devoid of SGAs content. It was found that highest percentage of SGAs present in n-butanol fractions, followed by DCM fractions, and mother extract. Further, SGAs containing extract/fraction were subjected to *in vitro* bioactivities with the remaining fractions for ultimate selection of enriched bioactive fractions. Fractions rich in secondary metabolites, including phenolic and flavonoids have antioxidant activities due to their redox properties. The n-butanol fraction of *S. nigrum* (leaves and berries) has strong antioxidant activity among other fractions against all free radical investigated. The DPPH radical is widely used in assessing free radical scavenging activity because of ease of reaction. Maximum DPPH scavenging activity was 75.23 and 78.34% for leaves and berries respectively, observed at concentration 125 μg/mL while that of control, ascorbic acid, was 83.42%. DPPH scavenging potential of fractions was increased with respect to dose of the extract upto 100 μg/mL. Further increment of fraction concentration (upto 125 μg/mL) resulting to no increment of DPPH scavenging potential (Figure 5C).

The total phenolic and flavonoids content of mother extract and fractions of *S. nigrum* was calculated from the calibration curve (*R*^2^ = 0.998) gallic acid and (*R*^2^ = 0.999) rutin. As their free radical scavenging ability is facilitated by their hydroxyl groups, the total phenolic concentration could be used as a basis for rapid screening of antioxidant activity (Chandini and Ganesan, 2008). Flavonoids suppress the reactive oxygen formation, chelate trace elements involved in free-radical production, scavenge reactive species and up-regulate and protect antioxidant defenses, (Figures 5A,B) (Agati et al., 2012).

### Cytotoxicity studies of SGAs enriched n-butanol fraction

Many reports suggest that the 5–50 mM of D-galactosamine were used for inducing the HepG2 cells death (Ariza and Paine, 1999; Liu et al., 2010). Further, it has been shown that the necrosis and apoptotic death are evident at moderate and high doses of D-galactosamine (Quintero et al., 2002). Hence, the cells were exposed to various concentrations of D-galactosamine (0–100 mM). The addition of 25 mM D-galactosamine to cells resulted in ~30.1% cell death and 59.9% elevation in reactive oxygen species. This moderate dose of 25 mM D-galactosamine was selected for further studies considering both modes of death i.e. necrotic and apoptotic cell death in Hep G2 cells. In separate studies, cells were then treated with various concentrations of SGAs n-butanol enriched fractions of *S. nigrum* berries (0–1,000 μg/mL) and leaves (0–1,000 μg/mL) for evaluation of cytotoxicity. The n-butanol fraction berries did not showed any toxicity up to 25 μg/Ml, however n-butanol leaves fraction did not produced any cytotoxicity upto 250 μg/mL.

### Cyto-protective effect of enriched fractions of *S. nigrum* against D-galactosamine cell death and generation of ROS

Exposure of the cells to 25 mM D-galactosamine, resulted in more cell death than the controls cells which was accompanied by the higher levels of reactive oxygen species. Treatment of cells with n-butanol fractions (berries) in the concentrations: 10 and 25 μg/mL showed the reduced cell death to 10.8 and 5.5%, respectively, when compared to cell death induced by 25 mM D-galactosamine alone (30.1%). While treatment of cells with n-butanol fraction (leaves) in the concentrations: 10 and 25 μg/mL showed reduction in cell death to 23.7 and 18.3%, respectively when compared to cell death induced by 25 mM D-galactosamine alone (30.1%).

Similarly, H_2_O_2_ treated cells on treatment with n-butanol fraction (berries concentrations): 10 μg/mL and 25 μg/mL showed reduced generation of ROS to 11.2 and 5.5%, respectively, as compared to ROS induced by H_2_O_2_ (500 μM) alone (59.9%). While treatment of cells with the n-butanol fraction of leaves in the concentrations: 10 and 25 μg/mL showed reduction in generation of ROS by 53.2 and 49.9%, respectively as compared to generation of ROS by H_2_O_2_. The protective effects of n-butanol fraction berries and leaves of *S. nigrum* were found to dose dependent.

### Bio-efficacy of enriched fractions of *S. nigrum* against D-galactosamine and generation of ROS induced leakage of cellular enzymes

Exposure of the cells to 25 mM D-galactosamine resulted in significant leakage of ALAT and ASAT by 7.14–5.78 folds, respectively. Treatment of cells with n-butanol fraction berries in the concentrations: 10 and 25 μg/mL showed reduced leakage of enzymes into extracellular medium by 2.80 and 1.33 folds, respectively when compared to enzyme leakage induced by 25 mM D-galactosamine alone (7.14 folds). While treatment of cells with n-butanol fraction of leaves in the concentrations: 10 and 25 μg/mL showed prevention in enzyme leakage by 4.73 and 3.68 folds, respectively compared D-galactosamine alone.

**Figure 6:**
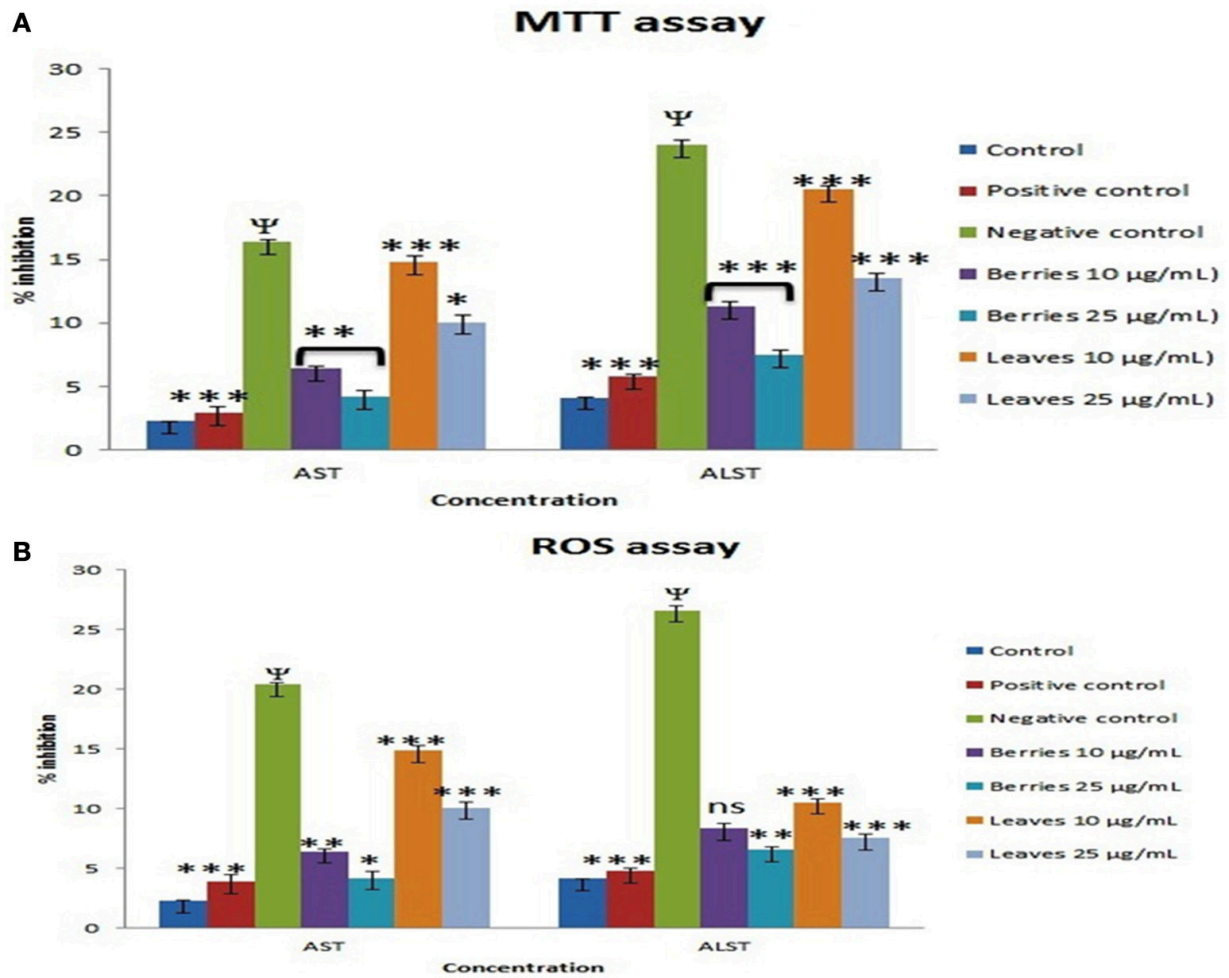
Effect of SGAs enriched fraction on hepatic marker enzyme by **(A)**, MTT and **(B)**, ROS. Each value is represented as mean ±SEM, *n* = 6, Ψ *P* < 0.01 when toxic control compare with control, ns, Non significance. ^*^P > 0.05, ^**^P < 0.01, ^***^P < 0.001 vs. toxic control, One way ANOVA followed by Dunet's test.

Treatment of cells with the n-butanol fraction of berries in the concentrations: 10 and 25 μg/mL showed reduced intracellular enzyme leakage by 2.73 and 1.4, respectively as compared to ROS induced by H_2_O_2_ (500 μM) alone (5.78 folds). While treatment of cells with n-butanol fraction leaves in the concentrations: 10 and 25 μg/ml showed reduced in enzyme leakage into culture medium by 4.94 and 3.99 folds, respectively as compared to enzyme leakage observed following treatment of H_2_O_2_ (500 uM) alone (5.78 folds). The protective effects of SGAs n-butanol enriched fractions from berries and leaves of *S. nigrum* were found to dose dependent (Figures 6A,B).

## Discussion

The approach that helps to explore the real therapeutic value of the natural pharmacotherapeutic agents and systematize the dosage schedule on evidence-based findings has become a fashionable trend. *S. nigrum* is widely used folk medicine for the treatment of hepatic diseases. Traditionally, the raw materials of *S. nigrum* (berries and leaves) were extracted by boiling water and administered orally. In spite of its tremendous therapeutic potential on liver disorders, complete chemical signature is unknown and thus it is the latent hazardous factor of administrating as herbal medicine. Therefore, we employed a fractionation process, which deployed to identify the lead druggable candidate from a given phytochemical matrix (Katiyar et al., 2012).

The phytoconstituents mostly effective in *S. nigrum* plant or from their family solanaceae are steroidal glycosides. Polar and medium polar metabolites often exhibit low retention on C18 columns potentially causing rise to effect of ion suppression. However, UPLC methods will allow high sample throughput and narrow peak shapes which will enable us to report results quickly and to achieve superior assay of sensitivity. This type of methods increase the sensitivity and decrease influential matrix effect. Using the gradient elution program mentioned in the methodology section, solasonine, solamargine, and solasodine elutes in a very sharp peak after ~1.5 min (Figure 3A). Among the three targeted metabolites, solasodine and solamargine were eluted at ~1.70 min but due to having different transition these are being easily quantified and detected.

A fast, accurate and proficient UPLC-ESI-MS/MS method was used for the quantitative analysis of the bioactive substances in *S. nigrum*. The simultaneous determination of bioactive compounds is a difficult task because of the complexity of the matrix and differences in the variation of the components. We have developed a rapid UPLC-ESI-MS/MS method for the simultaneous quantitative determination of major metabolites viz. SGAs plant fractions, including solasonine, solamargine, and solasodine. Using the new method, a typical experimental run was completed in about 5 min, which, coupled with the relatively low flow rate of 0.4 mL/min, resulted in a noticeable reduction in solvent consumption, and thus environmental impact compared to conventional HPLC systems. Because the new method does not require time-consuming pre-concentration steps, even large sample sets can be analyzed in limited period of time (Chen et al., 2015). However, the quality control parameters, we carried out HPTLC by exploring different solvents for fractionation. The MS/MS spectrum of solasodine, solamargine and solasonine are shown in Figure 3, together with the sum formulas and some likely structures of the fragments. Mass error in these experiments is < ±2 ppm where mass for internal lock was not used. The relative intensity of fragments of targeted molecule, generated in a triple quadrupole and an ion trap are usually different for a number of reasons. Argon as collision energy and Helium as collision was used. In IntelliStart (Waters, India) program, instrument automatically optimized the mass conditions for these said metabolites. Fragmentation based on ion ration as compared to parent ions were targeted and conditions were optimized. To determine the structure of fragment ions is not an easy task and very likely the reality will be more complex than proposed here.

HPTLC analysis of different fractions along with quantification of major metabolites viz. steroidal glycosides namely solasonine, solamargine and its aglycone part solasodine, *in vitro* antioxidant, phenolic, and flavonoid activities as reported strong correlation between the phenolic content in all the fractions and the DPPH free radical scavenging activity (Jain S. et al., 2013). The scavenging activity of DPPH free radicals had been used extensively to determine the antioxidant power of bioactive natural products (Moukette et al., 2015). Since antioxidant rich plant extracts serves as sources of nutraceuticals that reduce the oxidative stress and therefore prevent or slow down the degenerative diseases (Reddy and Grace, 2016).

Previous reports suggested that enrichment of phenolic compounds within plant extracts is correlated with their enhanced antioxidant activity and capability to scavenge free radicals (Arullappan et al., 2015).

Phenolic compounds are having a diverse group of molecules with an aromatic ring containing one or more hydroxyl groups responsible for its antioxidant potential. The main properties of phenolic compounds are to scavenge free radicals, donate hydrogen, chelate metal ions, and quench singlet oxygen (Shazila et al., 2010).

*S. nigrum* is one of the important medicinal plants that has been used traditionally for the treatment of various disease (Abbas et al., 2014). The antioxidant potential of n-butanol fraction and hydroalcoholic extract was shown to be correlated to polyphenol content, indicating that phenolic compounds are probably responsible for this activity. Thus, the main mode of hepatoprotective activity through preventing oxidative damage and scavenging free radicals (Indira et al., 2014). *S. nigrum* as whole herb is diuretic, antiperiodic, febrifuge, antiphlogistic, emollient, purgative, and sedative (Liu et al., 2016). In this experiment, leaves and berries have been selected to explore its hepatoprotective potentials.

n-butanol fractions of hydroalcoholic extracts of *S. nigrum* have shown significant antioxidant activity in DPPH radical scavenging activity, and in support of this activity, the total phenolic content was estimated.

Our study conclude that n-butanol fraction to be enriched with steroidal glycosides potential as reasonable mechanism for this fact may be that the nature of glycosides shows solubility-activity relationship having lipophilic and hydrophilic nature and since n-butanol being partially miscible in water, proper organic-to-aqueous volume ratios was maintained. Finally we investigated the hepatoprotective activity of the steroidal glycoside n-butanol enriched fraction of plant using D-galactosamine induced toxicity which shows significant (*p* < 0.01 and *p* < 0.05) liver protection against the hepatic injuries caused by the d- galactosamine in Hep G2 cell lines. D-galactosamine selectively depletes uridine nucleotides in the liver, inhibits RNA synthesis in hepatocytes and potentiates the acute toxicity (Lekic et al., 2011). These observations provided a basis for the potential hepatoprotective agent. Therefore, the overall hepatoprotective effect of *S. nigrum* is probably due to its antioxidant potential, or due to its stabilizing action of hepatocellular membrane or to maintain level of free radical enzymes at near to normal status.

## Conclusion

This study provides a wide-range of data so as to support some extent the use of SGAs enriched fraction from *S. nigrum* aerial part as phytopharmaceuticals to make advancement in evidence-based alternative medicine for the management of hepatics diseases. In this study, for the first time it was proposed a simple and efficient methods using UPLC-ESI-MS/MS as well as HPTLC analytical method for solasonine, solamargine, and its aglycone solasodine, which found to be excellent for routine analysis of SGAs, further it can become a pioneer in quality control aspect of *S. nigrum* and its herbal formulations.

## Author contributions

KC: Study design, literature review, experimental studies, data collection, and manuscript preparation. SP: Conceived and designed the experiment and expert comment. WK: Analysis and interpretation and manuscript preparation. SA: Conceived and designed the experiment, analyse and interpretation of data, and critical evaluation of manuscript.

### Conflict of interest statement

The authors declare that the research was conducted in the absence of any commercial or financial relationships that could be construed as a potential conflict of interest.
